# Estradiol-dependent axogenesis and *Ngn3* expression are determined by XY sex chromosome complement in hypothalamic neurons

**DOI:** 10.1038/s41598-020-65183-x

**Published:** 2020-05-19

**Authors:** Carla Daniela Cisternas, Lucas Ezequiel Cabrera Zapata, Franco Rafael Mir, María Julia Scerbo, María Angeles Arevalo, Luis Miguel Garcia-Segura, María Julia Cambiasso

**Affiliations:** 1 Instituto de Investigación Médica Mercedes y Martín Ferreyra, INIMEC-CONICET-Universidad Nacional de Córdoba, Córdoba, Argentina; 2 Departamento de Biología Bucal, Facultad de Odontología -Universidad Nacional de Córdoba, Córdoba, Argentina; 3 Instituto Cajal, CSIC, Madrid, Spain; 4 Ciber de Investigación Biomédica en Red de Fragilidad y Envejecimiento Saludable (CIBERFES), Instituto de Salud Carlos III, Madrid, Spain

**Keywords:** Cell biology, Neuroscience

## Abstract

Hypothalamic neurons show sex differences in neuritogenesis, female neurons have longer axons and higher levels of the neuritogenic factor neurogenin 3 (Ngn3) than male neurons *in vitro*. Moreover, the effect of 17-β-estradiol (E2) on axonal growth and Ngn3 expression is only found in male-derived neurons. To investigate whether sex chromosomes regulate these early sex differences in neuritogenesis by regulating the E2 effect on Ngn3, we evaluated the growth and differentiation of hypothalamic neurons derived from the “four core genotypes” mouse model, in which the factors of “gonadal sex” and “sex chromosome complement” are dissociated. We showed that sex differences in neurite outgrowth are determined by sex chromosome complement (XX > XY). Moreover, E2 increased the mRNA expression of Ngn3 and axonal length only in XY neurons. ERα/β expressions are regulated by sex chromosome complement; however, E2-effect on Ngn3 expression in XY neurons was only fully reproduced by PPT, a specific ligand of ERα, and prevented by MPP, a specific antagonist of ERα. Together our data indicate that sex chromosomes regulate early development of hypothalamic neurons by orchestrating not only sex differences in neuritogenesis, but also regulating the effect of E2 on Ngn3 expression through activation of ERα in hypothalamic neurons.

## Introduction

Many of the known sex differences found in mammalian brain networks and behavior are organized during early development and a compelling body of evidence has effectively linked many of these sex differences to the effect of a developmental exposure to gonadal testosterone or its metabolite 17-β-estradiol (E2)^1^. E2 has been shown to stimulate neuritogenesis at the time when neural circuits become functional in the brain^2–5^. More recently, the effect of sex chromosome-dependent factors during development on the sexual differentiation of the brain has been studied using the mouse model known as Four Core Genotypes (FCG)^6^. This animal model allows the evaluation of any sexually differentiated endpoint between XY and XX mice with testes or ovaries, i.e. XY male (XYM) or female (XYF) and XX male (XXM) or female (XXF) mice^7,8^. Using the FCG model, the early developmental effect of sex chromosome complement on sex differences in the brain has been demonstrated in the number of mesencephalic neurons immunoreactive for tyrosine hydroxylase^9^, as well as in the expression of the enzyme aromatase in the anterior amygdala^10,11^ and the neuritogenic factor neurogenin 3 (Ngn3) in hypothalamic neurons^12^. These sex differences were found in primary neuronal cultures or tissue obtained from FCG mice of embryonic age (E) 14–16 meaning that they cannot be attributed to the prenatal peak in gonadal testosterone occurring at E17–18^13,14^.

*Ngn3*, an autosomal gene involved in the signaling pathway of Notch^15^, is required for neuronal subtype specification in the ventromedial hypothalamus^16^, and it has been implicated in the degree of dendritic arborization in hippocampal neurons^17,18^ and in the rate of hypothalamic neuronal development^12^. Typically, hypothalamic neuronal cultures prepared with E14–16 female embryos develop faster and with longer axons than male neurons and these sex differences depend on a higher expression of *Ngn3* in female neurons than in male cultures^12^. The sex difference in *Ngn3* is dependent on the sex chromosome complement since XX male or female hypothalamic neurons express higher levels of *Ngn3* than XY cultures^12^. In addition, E2 increases *Ngn3* expression^12^ and axonal growth^12,19,20^ only in male neurons, counterbalancing the sex differences in neuritogenesis during early hypothalamic development.

To further investigate the effect of sex chromosomes on sex differences in the E2-effect and neuritogenesis, we evaluated the growth and differentiation of hypothalamic neurons derived from FCG in cultures with or without the hormone. In addition, we tested the hypothesis that sex chromosomes regulate early sex differences in neuritogenesis by regulating the E2 effect on Ngn3.

## Results

### Female hypothalamic cultures from MF1 and CD1 mice express higher levels of Ngn3 than male cultures

**Figure 1:**
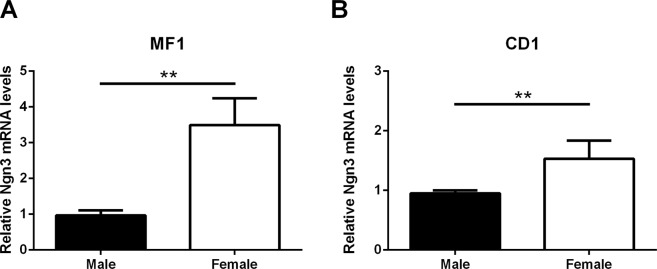
Female neuronal cultures express higher levels of Ngn3 than male cultures at embryonic day 14. Hypothalamic neuronal cultures of (**A**) MF1 and (**B**) CD1 mice. Bars indicate mean + SEM. n = 11 independent cultures for MF1 cultures and n = 6 for CD1 cultures. **P ≤ 0.01.

In agreement with previous studies in CD1 mice^12^, we found that female hypothalamic neurons from MF1 mice expressed higher levels of *Ngn3* than male cultures at E14 (Fig. 1; two- tailed T test: t_20_ = 3.331, *P* = 0.003 for MF1 cultures; two- tailed T test: t_10_ = 4.316, *P* = 0.002 for CD1 cultures).

### Sex differences in neurite outgrowth are determined by sex chromosome complement

**Figure 2:**
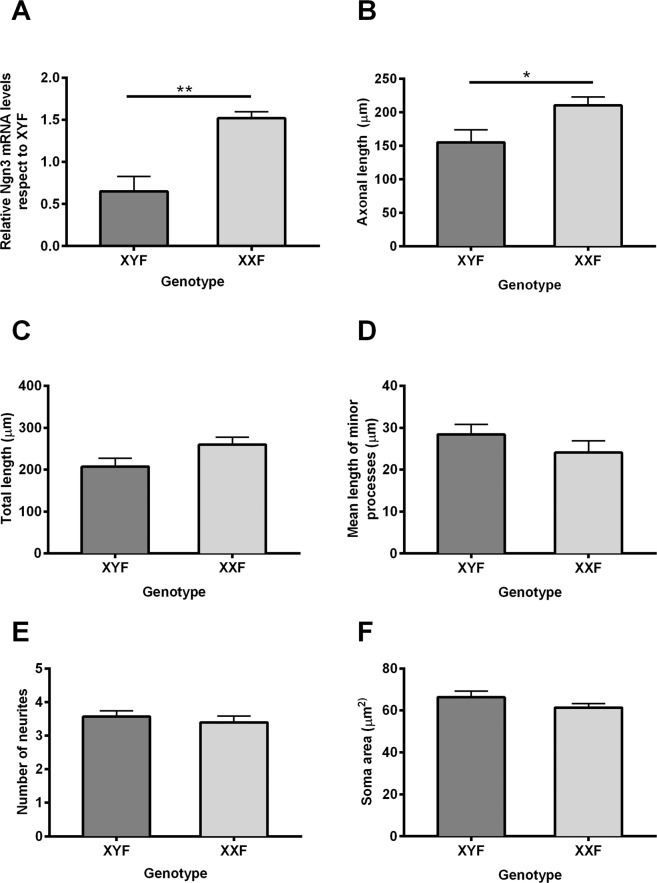
Sex chromosome complement determines sex differences in neuritogenesis. (**A**) Relative Ngn3 mRNA expression, (**B**) axonal length, (**C**) total length, (**D**) length of minor processes, (**E**) number of neurites and (**F**) soma area of XY female (XYF) and XX female (XXF) hypothalamic neurons. XXF neurons present higher levels of the neuritogenic factor Ngn3 as well as longer axonal length. Data are mean ± SEM. n = 5–8 independent cultures for each genotype. *P < 0.05; **P < 0.01.

**Figure 3:**
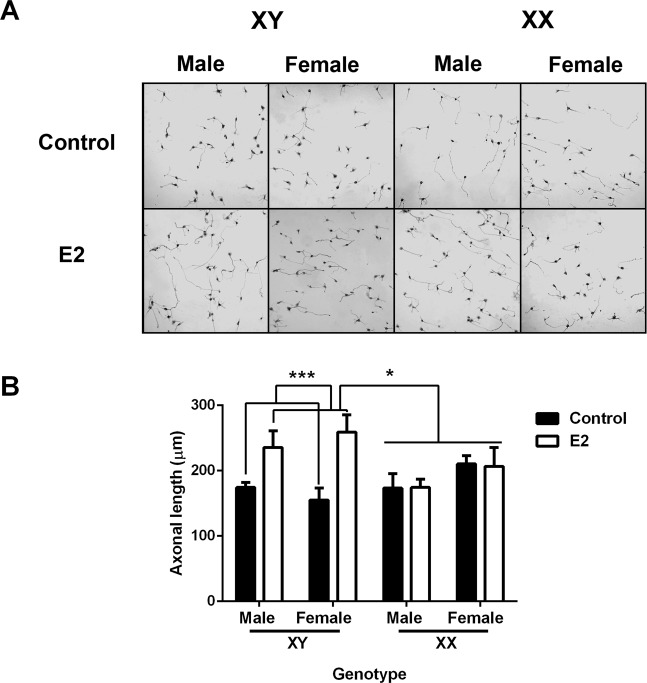
Sex chromosome complement determines the neuritogenic effects of estradiol in hypothalamic neurons. (**A**) Representative images of 2 DIV hypothalamic neurons derived from the “four core genotype” mouse model treated with or without 17-β-estradiol (E2), (**B**) E2 significantly increased the axonal length only in XY male and female (XYM and XYF) neuronal cultures. Data are mean ± SEM. n = 5–7 independent cultures for each genotype. *P < 0.05; ***P < 0.001.

Our previous results indicate that male and female hypothalamic neurons from CD1 mouse cultures present morphologic sex differences in neurite outgrowth starting at 2 days *in vitro* (DIV) and these sex differences are maintained through 7 DIV^12^. Female neurons present longer axons and higher expression of Ngn3 than male neurons^12^. In order to evaluate the direct role of sex chromosome complement in the described sex differences in neuronal maturation, we analyzed different morphological parameters of cellular shape and Ngn3 expression in hypothalamic neurons of XYF and XXF cultures of MF1 mice after 2 DIV (Fig. 2). The comparison of XY and XX female neurons allowed us to exclude any effects of gonadal testosterone produced before E14. As expected, XXF neurons expressed higher levels of *Ngn3* than XYF neurons (two- tailed T test: t_11_ = 3.681, *P* = 0.004; Fig. 2A). Regarding to morphological parameters of neuronal development, XXF neurons presented longer axons than XYF neurons (two- tailed T test: t_8_ = 2.487, *P* = 0.04; Figs. 2B and 3A). There were no effects of sex chromosomes on total neuritic length, mean length of minor processes, number of neurites, or soma area (Fig. 2C–F).

### Estradiol increases axonal length and Ngn3 mRNA expression only in XY neurons

We next asked whether sex chromosome complement determines the effect of E2 on axonal growth and *Ngn3* expression in male and female cultures of FCG MF1 mice. Three-way ANOVA indicated a significant effect of treatment (F_1,29_ = 6.98; *P* = 0.013) and genotype by treatment interaction (F_1,29_ = 7.466; *P* = 0.01). E2 significantly increased axonal length in XY neurons, irrespectively if they were male or female (*P* < 0.001, Fig. 3). In contrast, E2 did not affect this parameter in XX neurons.

**Figure 4:**
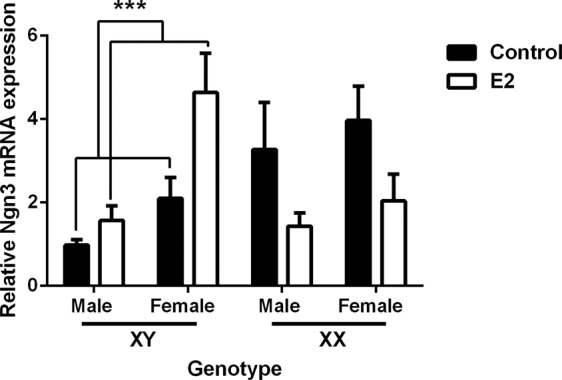
Estradiol increases the mRNA expression of Ngn3 only in XY chromosome complement. 17-β-estradiol (E2) significantly increased Ngn3 expression only in XY male and female (XYM and XYF) neuronal cultures. Data are mean ± SEM. n = 4–7 independent cultures for each genotype. ***P < 0.001.

Regarding the effect of E2 on *Ngn3* expression, a three-way ANOVA indicated a significant effect of gonadal sex (F_1,45_ = 10.90; *P* = 0.0019) as well as genotype by treatment interaction (F_1,45_ = 90.20; *P* = 0.004). E2 treatment significantly increased the relative expression of *Ngn3* mRNA in XYM and XYF neurons (*P* = 0.001, Fig. 4). Importantly, the effect of E2 on XY neurons abolished the sex chromosome-dependent difference with XX control cultures (XY + E2 vs. XX control cultures: *P* = 0.71).

### XY regulates estrogen receptor expression in hypothalamic neurons

**Figure 5:**
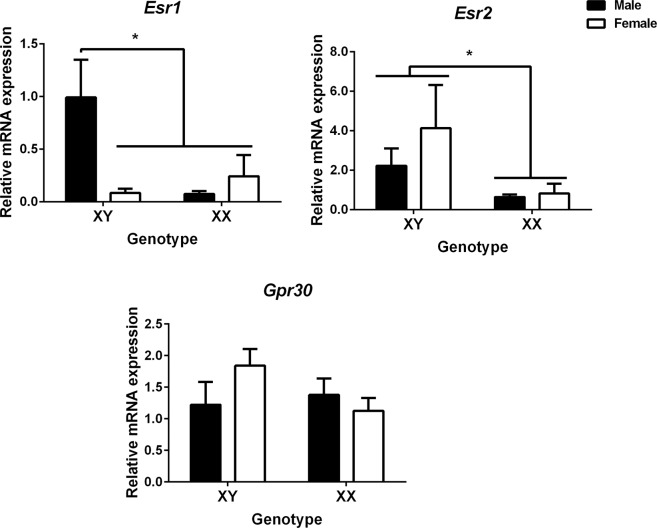
Sex chromosome complement determines the mRNA expression of ERα and ERβ in hypothalamic neurons. mRNA expression of ERα (*Esr1*), ERβ (*Esr2*) and G protein-coupled estrogen receptor 1 (GPER, *Gpr30*) in hypothalamic neurons of MF1 FCG cultures. Data are mean ± SEM. n = 4–6 independent cultures. *P < 0.05.

To determine whether neuritogenic effects seen in hypothalamic neurons depend on a differential availability of estrogen receptors (ER), we assessed the mRNA levels of ERα (*Esr1*), ERβ (*Esr2*) and G protein-coupled estrogen receptor 1 (GPER, *Gpr30*) in cultures of FCG MF1 mice. As shown in Fig. 5, two-way ANOVA found a significant interaction of sex chromosomes and gonadal sex for *Esr1* expression (F_1,18_ = 6.02; *P* = 0.024). XYM neurons express higher levels of *Esr1* than XYF or XX (male or female) neurons (*P* < 0.05). In addition, *Esr2* expression was determined by sex chromosome complement (F _1, 17_ = 6.06; *P* = 0.024; Fig. 5). XY male or female neurons express higher levels of *Esr2* mRNA than XX male or female neurons (*P* < 0.05). The expression of *Gpr30* did not differ among groups.

### Neuritogenic effect of estradiol on *Ngn3* expression depends on ERα

**Figure 6:**
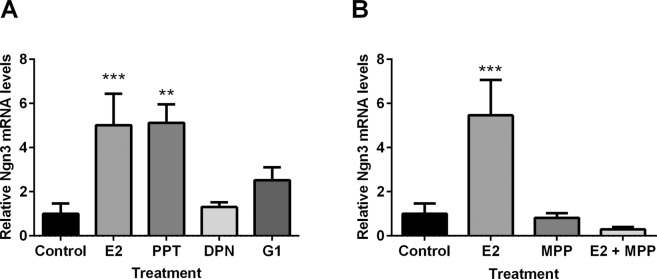
ERα mediates the neuritogenic effect of estradiol on Ngn3 expression. (**A**) The ERα agonist PPT mimicked the effect of estradiol (E2) on Ngn3 expression while (**B**) the selective ERα antagonist MPP abolished the E2-induced increase in Ngn3 expression in male neuronal cultures from CD1 mice. Data are mean ± SEM. N = 3–7 independent cultures. **P < 0.01; ***P < 0.001.

In the analyses above, ERα mRNA expression was higher in XYM neurons, while ERβ mRNA expression was higher in XY male and female neurons. These results suggest that E2 dependent *Ngn3* expression was mediated by either one of these ERs. In order to further identify the subtype of ER involved in the effect of E2 on *Ngn3* expression, we used wild type CD1 male hypothalamic cultures. Male neuronal cultures were incubated with either E2 or specific agonists for ERα, ERβ, or GPER. One-way ANOVA found a significant effect of treatment (F_4,29_ = 5.35; *P* = 0.002; Fig. 6A). As expected, E2 significantly increased *Ngn3* expression (*P* = 0.0006) and this effect was mimicked by the ERα agonist PPT (control cultures compared to PPT-treated cultures: *P* = 0.0028). Treatment with the ERβ agonist DPN or with the GPER agonist G1 did not significantly affect *Ngn3* expression (*P* > 0.05). To further demonstrate that ERα is involved in the E2-induced upregulation of *Ngn3* in male cultures, hypothalamic cultures were co-treated with E2 and the ERα antagonist MPP (Fig. 6B). One-way ANOVA found a significant effect of treatment (F_3,21_ = 6.19; *P* = 0.003): as expected, E2 upregulated *Ngn3* expression (*P* = 0.0007) and this effect was blocked when MPP was added in combination with E2 (*P* = 0.64). MPP alone did not affect the expression of the neuritogenic gene (*P* > 0.05).

## Discussion

Previously, we have demonstrated a sex difference in neuritogenesis and *Ngn3* expression in hypothalamic cultures of E14 CD1 mouse^12^. In order to corroborate this sex difference in the FCG mouse model, we used MF1 WT mice to prepare primary neuronal cultures since this strain is the genetic background of our FCG mouse colony. In agreement with our previous data in CD1 mice, female neuronal cultures from MF1 embryos expressed higher levels of *Ngn3* mRNA. Therefore, although the use in the present study of two different mice strains, MF1 and CD1, is not exempt of some limitations, our findings suggest that both strains have similar characteristics concerning *Ngn3* expression in the embryonic hypothalamus, and that the FCG MF1 mouse is a valid model to study the mechanism of hormonal regulation of neuritogenesis *in vitro*.

Here, we found that sex chromosomes orchestrate sex differences in neuritogenesis in hypothalamic neurons. In concordance with our previous results^12^, we have shown that XXF neurons present longer axons than XYM; furthermore, our present results demonstrate that XY hypothalamic neurons respond to E2 increasing axonal length regardless of their gonadal status. This estrogenic effect was concomitant with enhanced *Ngn3* expression in XY cultures, thereby enhanced neuritogenesis. We also found that E2 and the ERα agonist PPT increased *Ngn3* expression in male hypothalamic neurons. In addition, the ERα antagonist MPP completely blocked the effect of E2 on *Ngn3* expression. Remarkably, our present results are in agreement with previous findings^21,22^, indicating that the axogenic effect of E2 in male hypothalamic neurons rely on an ERα-dependent mechanism.

Multiple studies have investigated the neuritogenic effect of E2, which is differentially exerted depending on the brain region^23–25^, sex^19,24^, and age of the donor neural tissue^19,20,24^. However, few studies have taken into account neuritogenesis prior to the organizational actions of gonadal hormones^26^. Previous work from our laboratory has demonstrated sex differences in neuritogenesis in primary neuronal hypothalamic cultures prepared before the peak of testosterone production by fetal testis^12^. Female neurons show increased axogenesis than male neurons suggesting cell-autonomous actions of sex chromosomes. In the present study, we found that female neurons bearing XX sex chromosome complement show higher axonal length than female neurons with XY complement (XX > XY). Hormonal treatment increased axon length only in XY neurons (irrespectively of gonadal sex), resulting in the reversion of sex differences in neuritogenesis (XX < XY) by a mechanism that also depends on genetic factors.

Sex differences in neuritogenesis have been related to sex differences in the expression of the proneural gene *Ngn3* (located in autosome 10), not only in the developing hypothalamus^12^ but also in the hippocampus^27^. Indeed, a down-regulation of *Ngn3* expression in hypothalamic cultures decreased neuronal maturation, axonal length, and the proportion of cells with branched neurites in female cultures, but not in male cultures^12^. Moreover, Ngn3 has also been involved in the neuritogenic actions of E2 in developing hippocampal neurons^28^. Previously, we showed that sex chromosomes generate the sex difference in *Ngn3* expression in hypothalamic neurons^12^. Our present results corroborate these findings and extend the role of sex chromosomes to the E2 effect on *Ngn3* expression in hypothalamic neurons. E2 enhanced *Ngn3* levels only in neurons from XY embryos whether the embryos had ovaries or testes. In contrast, no increased level of *Ngn3* by E2 was found in cultures from embryos with XX sex chromosome complement. One possible explanation for the different response to E2 is a varied enrichment of classical and non-classical ERs in hypothalamic neurons according to the sex chromosome complement. For example, our previous results using FCG mice indicate that the amygdala in XY males and females express higher levels of ERβ (*Esr2*) than XX mice, both *in vivo*^10^ and *in vitro*^11^, whereas ERα (*Ers1*) is regulated by both sex chromosome complement and gonadal phenotype^11^. Here we found that in the developing hypothalamus, *Esr1* and *Esr2* expression was also dependent on XY sex chromosome complement. XYM neurons express higher levels of *Esr1* and *Esr2* than XX (male or female) neurons. Moreover, *Esr2* was also increased in XYF neurons. The expression of the non-classical ER, Gpr30, was not subjected to sex chromosome influence. These findings open up the question of ERα/β involvement on E2-dependent regulation of *Ngn3* expression. To unravel this possibility, we evaluated several compounds that act as agonists or antagonists of classical steroid action. PPT, an ERα agonist, imitates the effect of E2 on *Ngn3* expression. By contrast, DPN and G1, selective agonists of ERβ and GP30‐dependent mechanisms, respectively, were not able to imitate the effect of E2 on *Ngn3*. Furthermore, MPP, a selective antagonist of ERα‐mediated transcription, was able to antagonize the effect of E2. Given that ER expression was dependent on XY sex chromosome complement; all these findings suggest that sex chromosome genes might primarily orchestrate the effectiveness of E2 effects by regulating the expression of ERα.

The difference in X- and Y- gene dosage resulting from the inheritance of XX vs. XY sex chromosome complement is one of the mechanisms producing sex differences in non-gonadal tissues^8^. Moreover, sex chromosomes may also counteract the effects of each other, reducing rather than producing sex differences in phenotype^29^. In contrast to the small and gene-poor Y chromosome (containing mostly genes related to testis determination and fertility), the X chromosome is much larger and encodes genes with a much broader range of functions^30,31^. Interestingly, many of these genes are involved in brain development^32^ and their mutations produce X-linked neurological diseases that are more harmful in males than in females^33^. Among the X-linked genes associated with neuronal growth and differentiation, there are some that escape X-inactivation^34^ and encode proteins related to chromatin remodeling, such as the histone demethylases *Kdm6a* and *Kdm5c*^35,36^. In the developing hypothalamus, these genes could potentially mediate the regulation of autosomal genes such as *Ngn3*, *Esr1* and *Esr2* in a sex dependent manner. Particularly, *Kdm6a* and *Kdm5c* are expressed in the brain^37^ and show higher levels of expression in XX vs. XY mice^37,38^, both before and after gonadal differentiation^39^. Moreover, the inhibition of Kdm5c in primary mammalian neurons impairs dendritic morphogenesis^35^, suggesting that it may regulate the autosomal gene expression involved in neuritogenesis. Unfortunately, the use of unsexed cultures in this study^35^ limits the interpretation of sex differences. Other gene that escapes X-inactivation in mice is *Mid1*^40^, a microtubule-associated protein that influences microtubule dynamics^41^. In polarized neurons, the endogenous *Mid1* expression is mainly located in the soma and axon. Moreover, silencing *Mid1* in developing neurons promotes axon growth and branch formation, resulting in a disruption of callosal axon projections in the contralateral cortex^42^. The male-specific effect of E2 on axonal growth and Ngn3 expression could be mediated through a downregulation of Mid1 expression. Based on this evidence, the most likely interpretation is that one or more X-linked genes may be contributing to a sex-specific effect of E2 in male neurons by regulating the expression of ERs, and thus influencing the sensitivity of the developing hypothalamus to sex steroid hormones.

Taken together, our results suggest a direct role of sex chromosomes in the development of sex differences in neuronal differentiation and maturation via an ERα-dependent mechanism. The scenario is likely to be strengthened in a region such as the ventromedial hypothalamus which contains a highly heterogeneous population of ERα-expressing neurons with different projections and sex-related functions^43–45^. The sex differences in neuritic development may establish the organization of hypothalamic neuronal projections to ensure precise structural and functional patterning, which is crucial for proper circuit formation of sex-related behaviors. The developing hypothalamus selectively expresses the steroidogenic factor 1 (SF-1) around E14^46^; this event is paramount for the proper organization of the VMH^47,48^. Interestingly, Ngn3 promotes the development of ventromedial SF1 neurons^16^, and given the role of sex chromosome complement in regulating hormonal and non-hormonal expression of Ngn3, these results suggest that sex chromosomes might participate in the generation of neuronal populations controlling sexual development and reproduction in mice. Since the E2 effect on Ngn3 expression in XY neurons abolished basal differences with control XX neurons, the final result might be the compensation of sex differences in Ngn3 expression which would ensure equivalent specification of SF1 neurons in males and females during brain development.

Sexual differentiation of the brain has long been considered a hormonally driven process, in great part, due to multiple evidence coming from the field of neuroendocrinology. Indeed, the classical hypothesis of hormonal sexual differentiation has effectively explained many of the known sex differences in the brain and other non-gonadal tissues. The hypothalamus, a major brain region controlling sexual and reproductive functions is a main example of a brain region considered predominantly, or exclusively, under hormonal control during development. However, there is evidence of sex chromosome complement playing a role in the organization of sex differences in the hypothalamus^49–51^. We recently demonstrated gonadal hormone-independent sex differences in GABAA receptors in hypothalamic cultures of rat embryos. Specifically, these effects were observed before the *in utero* surge in gonadal hormones and thus were independent of hormonal treatment of cultures^52^. Taken together, all these studies provide evidence to rethink the classic model and to incorporate the role of sex chromosome complement, not only as a primary orchestrator of early sex differences in the development of hypothalamic neurons, but also as gating the action of sex steroids at this early stage of development. This action might be relevant for the sexual differentiation hypothesis as it contributes to the integration of multiple, separate sex-biasing factors.

## Methods

### Animals

The embryos used in this study were obtained from CD1 mice raised at the Instituto Cajal (Madrid, Spain) or at the Instituto Ferreyra (Córdoba, Argentina). The researcher who did the experiments in Madrid and Córdoba was always the same (C.D.C.). Some experiments also involved transgenic mice obtained from MF1 mice of the Four Core Genotypes (FCG) mouse model, bred in our colony at the Instituto Ferreyra. This MF1 outbred colony was a gracious donation from Dr. Paul Burgoyne (National Institute for Medical Research, London UK). The embryos used in FCG cultures were produced by breeding transgenic MF1 XYM mice with wild-type MF1 females (Harlan Laboratories Inc. US). Mice were maintained on a 12:12 light dark cycle with *ad libitum* access to food and water. All procedures involving the animals used in this study were approved by the animal care and use committees at our institutions (Animal Care and Use Committee, CICUAL-IMMF, INIMEC-CONICET-UNC; Comité de Ética de Experimentación Animal, Instituto Cajal) and by the Consejeria del Medio Ambiente y Territorio (Comunidad de Madrid, Ref. PROEX 200/14) and followed the National Institutes of Health Guide for the Care and Use of Laboratory Animals. Genotyping was performed as previously described in^10^. Timed pregnancies were established by pairing breeders within an hour of lights off and removing males the following morning after lights on. E1 was defined as the day of vaginal plug presence.

### Cell cultures

Embryos of E14 were used to prepare hypothalamic neuronal cultures as previously described in^12^. The embryos were classified according to the sex (by observation of the spermatic artery of the developing testes) and/or genotype (by PCR). The ventromedial hypothalamic region was dissected out and stripped off the meninges and the blocks of tissue were then mechanically dissociated to single cells after digestion for 15 min at 37 ^◦^C with 0.5% trypsin (Worthington Biochemicals, Freehold, NJ, USA). The medium was phenol red-free Neurobasal (Invitrogen, United Kingdom) to avoid “estrogen-like effects”^53^ and was supplemented with B-27, N-2, 0.043% L-alanyl-L-glutamine (GlutaMAX I), 0.15% glucose, 100 U/ml penicillin and 100 mg/ml streptomycin (Invitrogen). The cells were seeded on the surfaces of glass coverslips or plates that were coated with poly-L-lysine (1 µg/µl; Sigma-Aldrich, USA).

For the analyses of gene expression, cells were plated onto 6-well plates and after 3 days *in vitro* (DIV) the medium was replaced by Neurobasal without supplements for 2 hours. The neuronal cultures were then treated for 2 h with the following compounds alone or in combination: E2 (10^−10^ M; Sigma-Aldrich), the selective ERα agonist propylpyrazoletriol (PPT, 10^−7^ M; Tocris Bioscience, United Kingdom), the selective ERβ agonist dyarilpropionitrile (DPN, 10^−9^ M; Tocris), the GPER agonist G1 (10^−6^ M, Calbiochem), the selective ERα antagonist 1,3-Bis(4-hydroxyphenyl)−4-methyl-5-[4-(2-piperidinylethoxy)phenol]−1H-pyrazole dihydrochloride (MPP, 10^−7^ M; Tocris). The concentrations used were based on our previous results^11,22,28^. For morphometric analyses, neurons were seeded onto coverslips of 12 mm diameter (Assistant, Germany) and treated with of E2 (10^−9^ M, Sigma-Aldrich) for 2 DIV.

### RNA purification and qRT-PCR

Total RNA was isolated from neuronal cultures with illustra RNAspin Mini RNA isolation kit from GE Healthcare (Buckinghamshire, UK). Single strand cDNA was prepared from total RNA using the M-MLV reverse transcriptase (Promega Corp., Madison, Wisconsin) following the manufacturer’s instructions. Quantitative PCR (qRT-PCR) reactions were performed on an ABI Prism 7000 Sequence Detector (Applied Biosystems, Weiterstadt, Germany) using the TaqMan or Sybr Green Universal PCR Master Mix. TaqMan probes and primers for Ngn3 were Assay-on-Demand gene expression products (Applied Biosystems). Primer sequences for the control housekeeping gene 18S rRNA and ERs are described in Table 1. All primers were verified to amplify with an efficiency of 95–100% using 4-point calibration curves. Relative quantification of mRNA expression was determined with the ΔΔCt method using the average of XY-Sry male as calibrator group for FCG cultures or, the average of male cultures as a calibrator group for wild type cultures.

**Table 1:** Primer sequences used for gene expression assays.

Primer name	Forward sequence 5′−3′	Reverse sequence 5′−3′
Esr1	ATGAAAGGCGGCATACGGAAAG	CACCCATTTCATTTCGGCCTTC
Esr2	CCTGGTCTGGGTGATTTCGA	ACTGATGTGCCTGACATGAGAAAG
Gper1	TGCTGCCATCCAGATTCAAG	GGGAACGTAGGCTATGGAAAGAA
18S rRNA	CGCCGCTAGAGGTGAAATTCT	CATTCTTGGCAAATGCTTTCG

### Immunocytochemistry and morphometric analyses

After 2 DIV, the hypothalamic cultures were fixed by immersion in 4% paraformaldehyde with 0.12 M sucrose at 37 °C. After extensive rinses in phosphate-buffered saline solution (PBS), cultures were stained against β-tubulin class III (SDL.3D10). The protocol is described in detail elsewhere^54^. Briefly, on the first day cells were permeabilized with 0.2% Triton X-100 at room temperature followed by incubation in bovine serum albumin (BSA) and incubation in primary antibody against β-tubulin class III (1:400, Sigma-Aldrich, St. Louis, MO, USA) at 4 °C. On the second day, cells were rinsed in PBS and then incubated with anti-mouse biotinylated secondary antibody for one hour at room temperature. Reaction was magnified by incubation in Vectastain ABC immunoperoxidase reagent (Vector Laboratories, Burlingame, CA, USA), and visualized by reaction with 1.4 mM 3,3′-diaminobenzidine in phosphate buffer with H_2_O_2_. Cells were then dehydrated with serial dilutions of ethanol and mounted on glass slides for morphometric analysis.

Morphometric analyses were done using an image processor (Jandel Inc., Richmond, CA, USA) through an optical microscope (Carl Zeiss, Germany). The operator doing all morphometric analyses was blind to the genotype and treatment of the cell cultures. At 40X magnification, the operator evaluated all labeled neurons identified as a single cell from all other surrounding cells. Morphometric parameters of hypothalamic neurons *in vitro* have been previously described^24^ and follow those described for hippocampal neurons in culture^23,55^. According to these morphologic criteria, after 2 DIV most cells have a single long “major” process, thin and uniform in diameter, while several shorter “minor” processes also emerge from the soma^55^. Neurons with a major process 3 to 5 times longer than the rest of other processes were considered to have developed an axon (stage III of development). We recorded the total axonal length, soma area, length of minor processes, and the number of neurites per cell of 60 neurons for every experimental condition in each culture.

### Statistical analyses

Data are expressed as mean + SEM. The sex chromosome effect on the expression of Ngn3 in FCG MF1 or CD1 cultures, as well as the neurite outgrowth were evaluated using two-tailed independent t-tests. The neuritogenic effect of E2, sex chromosomes and gonadal sex in FCG cultures was evaluated using three-way ANOVA. Two-way ANOVA was used to evaluate the effect of sex chromosomes and gonadal sex on the expression of ERs. One-way ANOVA was used to evaluate the mechanism of E2 effect on Ngn3 expression in male cultures. ANOVA was followed by Fisher’s least significance difference (LSD) post hoc test when appropriate. A level of p < 0.05 was considered as statistically significant. The n for statistical analysis was the number of independent cultures that corresponds to the number of pregnant mothers in CD1 cultures. For cell cultures prepared from FCG mice, the number of independent neuronal cultures corresponds to the number of embryos of each genotype and treatment. The FCG embryos were obtained from at least 5 pregnant mothers.

## Data Availability

No datasets were generated or analyzed during the current study. RRID:AB_2314198, Sigma-Aldrich Cat# SDL.3D10. RRID:AB_2338504, Jackson ImmunoResearch Labs Cat# 115-035-062.

## References

[1] Lenz KM, McCarthy MM (2010). Organized for sex - steroid hormones and the developing hypothalamus. Eur. J. Neurosci..

[2] Arnold AP, Gorski RA (1984). Gonadal steroid induction of structural sex differences in the central nervous system. Annu. Rev. Neurosci..

[3] McEwen BS (1991). Steroid hormones as mediators of neural plasticity. J. Steroid Biochem. Mol. Biol..

[4] Carrer HF, Cambiasso MJ (2002). Sexual differentiation of the brain: genes, estrogen, and neurotrophic factors. Cell. Mol. Neurobiol..

[5] McCarthy MM (2008). Estradiol and the developing brain. Physiol. Rev..

[6] Arnold AP (2009). The organizational-activational hypothesis as the foundation for a unified theory of sexual differentiation of all mammalian tissues. Horm. Behav..

[7] Arnold AP (2017). A general theory of sexual differentiation. J. Neurosci. Res..

[8] Arnold AP (2019). Rethinking sex determination of non-gonadal tissues. Curr. Top. Dev. Biol..

[9] Carruth LL, Reisert I, Arnold AP (2002). Sex chromosome genes directly affect brain sexual differentiation. Nat. Neurosci..

[10] Cisternas C (2015). Sex chromosome complement determines sex differences in aromatase expression and regulation in the stria terminalis and anterior amygdala of the developing mouse brain. Mol. Cell. Endocrinol..

[11] Cisternas C, Cabrera Zapata L, Arevalo M, Garcia-Segura L, Cambiasso M (2017). Regulation of aromatase expression in the anterior amygdala of the developing mouse brain depends on ERβ and sex chromosome complement. Sci. Rep..

[12] Scerbo MJ (2014). Neurogenin 3 mediates sex chromosome effects on the generation of sex differences in hypothalamic neuronal development. Front. Cell. Neurosci..

[13] O’Shaughnessy PJ (1998). Fetal development of Leydig cell activity in the mouse is independent of pituitary gonadotroph function. Endocrinology.

[14] O’Shaughnessy, P. J., Baker, P. J. & Johnston, H. The foetal Leydig cell–differentiation, function and regulation. *Int. J. Androl*. **29**, 90–95; discussion 105-108 (2006). 10.1111/j.1365-2605.2005.00555.x 16466528

[15] Bertrand N, Castro DS, Guillemot F (2002). Proneural genes and the specification of neural cell types. Nat. Rev. Neurosci..

[16] Pelling M (2011). Differential requirements for neurogenin 3 in the development of POMC and NPY neurons in the hypothalamus. Dev. Biol..

[17] Salama-Cohen P, Arévalo M-A, Grantyn R, Rodríguez-Tébar A (2006). Notch and NGF/p75NTR control dendrite morphology and the balance of excitatory/inhibitory synaptic input to hippocampal neurones through Neurogenin 3. J. Neurochem..

[18] Simon-Areces J, Membrive G, Garcia-Fernandez C, Garcia-Segura LM, Arevalo M-A (2010). Neurogenin 3 cellular and subcellular localization in the developing and adult hippocampus. J. Comp. Neurol..

[19] Cambiasso MJ, Díaz H, Cáceres A, Carrer HF (1995). Neuritogenic effect of estradiol on rat ventromedial hypothalamic neurons co-cultured with homotopic or heterotopic glia. J. Neurosci. Res..

[20] Cambiasso, M. J., Colombo, J.A. & Carrer, H. F. Differential effect of oestradiol and astroglia-conditioned media on the growth of hypothalamic neurons from male and female rat brains. *Eur. J. Neurosci*. **12**, 2291–2298 (2000). 10.1046/j.1460-9568.2000.00120.x 10947808

[21] Gorosito SV, Cambiasso MJ (2008). Axogenic effect of estrogen in male rat hypothalamic neurons involves Ca(2+), protein kinase C, and extracellular signal-regulated kinase signaling. J. Neurosci. Res..

[22] Gorosito SV, Lorenzo AG, Cambiasso MJ (2008). Estrogen receptor alpha is expressed on the cell-surface of embryonic hypothalamic neurons. Neuroscience.

[23] Blanco G, Diaz H, Carrer HF, Beaugé L (1990). Differentiation of rat hippocampal neurons induced by estrogen *in vitro*: effects on neuritogenesis and Na, K-ATPase activity. J. Neurosci. Res..

[24] Díaz H, Lorenzo A, Carrer HF, Cáceres A (1992). Time lapse study of neurite growth in hypothalamic dissociated neurons in culture: sex differences and estrogen effects. J. Neurosci. Res..

[25] Lorenzo A, Díaz H, Carrer H, Cáceres A (1992). Amygdala neurons *in vitro*: neurite growth and effects of estradiol. J. Neurosci. Res..

[26] Reisert I, Pilgrim C (1991). Sexual differentiation of monoaminergic neurons–genetic or epigenetic?. Trends Neurosci..

[27] Ruiz-Palmero I (2016). Oestradiol synthesized by female neurons generates sex differences in neuritogenesis. Sci. Rep..

[28] Ruiz-Palmero I, Simon-Areces J, Garcia-Segura LM, Arevalo MA (2011). Notch/Neurogenin 3 Signalling is Involved in the Neuritogenic Actions of Oestradiol in Developing Hippocampal Neurones. J. Neuroendocrinol..

[29] Arnold, A. P. Conceptual frameworks and mouse models for studying sex differences in physiology and disease: Why compensation changes the game. *Exp. Neurol*. 1–8 10.1016/j.expneurol.2014.01.021 (2014). 10.1016/j.expneurol.2014.01.021 PMC4125548 24509348

[30] Graves JAM (2006). Sex chromosome specialization and degeneration in mammals. Cell.

[31] Wijchers PJ, Festenstein RJ (2011). Epigenetic regulation of autosomal gene expression by sex chromosomes. Trends Genet..

[32] Ropers H-H, Hamel BCJ (2005). X-linked mental retardation. Nat. Rev. Genet..

[33] Chiurazzi P, Schwartz CE, Gecz J, Neri G (2008). XLMR genes: update 2007. Eur. J. Hum. Genet..

[34] Carrel L, Willard HF (2005). X-inactivation profile reveals extensive variability in X-linked gene expression in females. Nature.

[35] Iwase S (2007). The X-linked mental retardation gene SMCX/JARID1C defines a family of histone H3 lysine 4 demethylases. Cell.

[36] Welstead GG (2012). X-linked H3K27me3 demethylase Utx is required for embryonic development in a sex-specific manner. Proc. Natl. Acad. Sci. USA.

[37] Xu J, Disteche CM (2006). Sex differences in brain expression of X- and Y-linked genes. Brain Res..

[38] Xu J, Deng X, Disteche CM (2008). Sex-specific expression of the X-linked histone demethylase gene Jarid1c in brain. PLoS One.

[39] Wolstenholme JT, Rissman EF, Bekiranov S (2013). Sexual differentiation in the developing mouse brain: Contributions of sex chromosome genes. Genes, Brain Behav..

[40] Yang F, Babak T, Shendure J, Disteche CM (2010). Global survey of escape from X inactivation by RNA-sequencing in mouse. Genome Res..

[41] Schweiger S (1999). The Opitz syndrome gene product, MID1, associates with microtubules. Proc. Natl. Acad. Sci. USA.

[42] Lu, T. *et al*. X-linked Microtubule-Associated Protein, Mid1, Regulates Axon Development. Proc. Natl. Acad. Sci. USA 110, (2013). 10.1073/pnas.1303687110 PMC3839708 24194544

[43] Chen R, Wu X, Jiang L, Zhang Y (2017). Single-cell RNA-seq reveals hypothalamic cell diversity. Cell Rep..

[44] Correa SM (2015). An estrogen-responsive module in the ventromedial hypothalamus selectively drives sex-specific activity in females. Cell Rep..

[45] Kim D-W (2019). Multimodal Analysis of Cell Types in a Hypothalamic Node Controlling Social Behavior. Cell.

[46] Ikeda Y, Shen WH, Ingraham HA, Parker KL (1994). Developmental expression of mouse steroidogenic factor-1, an essential regulator of the steroid hydroxylases. Mol. Endocrinol..

[47] Shinoda K (1995). Developmental defects of the ventromedial hypothalamic nucleus and pituitary gonadotroph in the Ftz-F1 disrupted mice. Dev. Dyn..

[48] Luo X, Ikeda Y, Parker KL (1994). A cell-specific nuclear receptor is essential for adrenal and gonadal development and sexual differentiation. Cell.

[49] Büdefeld, T., SA, Tobet, S. & Majdič, G. Gonadal hormone independent sex differences in steroidogenic factor 1 knockout mice brain. *Slov. Vet. Zb*. **47** (2010). PMC3163895 21887123

[50] Majdic G, Tobet S (2011). Cooperation of sex chromosomal genes and endocrine influences for hypothalamic sexual differentiation. Front. Neuroendocrinol..

[51] Grgurevic N, Büdefeld T, Spanic T, Tobet SA, Majdic G (2012). Evidence that sex chromosome genes affect sexual differentiation of female sexual behavior. Horm. Behav..

[52] Mir, F. R., Wilson, C., Cabrera Zapata, L. E., Aguayo, L. G. & Cambiasso, M. J. Gonadal hormone-independent sex differences in GABAA receptor activation in rat embryonic hypothalamic neurons. *Br. J. Pharmacol*. 10.1111/bph.15037 (2020). 10.1111/bph.15037 PMC7280008 32133616

[53] Berthois Y, Katzenellenbogen JA, Katzenellenbogen BS (1986). Phenol red in tissue culture media is a weak estrogen: implications concerning the study of estrogen-responsive cells in culture. Proc. Natl. Acad. Sci. USA.

[54] Cabrera Zapata LE, Bollo M, Cambiasso MJ (2019). Estradiol-Mediated Axogenesis of Hypothalamic Neurons Requires ERK1/2 and Ryanodine Receptors-Dependent Intracellular Ca2+ Rise in Male Rats. Front. Cell. Neurosci..

[55] Dotti CG, Sullivan CA, Banker GA (1988). The establishment of polarity by hippocampal neurons in culture. J. Neurosci..

